# How Far is the Spanish Snack Sector from Meeting the Acrylamide Regulation 2017/2158?

**DOI:** 10.3390/foods9020247

**Published:** 2020-02-24

**Authors:** Marta Mesias, Aouatif Nouali, Cristina Delgado-Andrade, Francisco J Morales

**Affiliations:** Institute of Food Science, Technology and Nutrition (ICTAN-CSIC), 28040 Madrid, Spain; anouali@ucm.es (A.N.); cdelgado@ictan.csic.es (C.D.-A.); fjmorales@ictan.csic.es (F.J.M.)

**Keywords:** acrylamide, potato crisps, dietary intake, exposure, consumers

## Abstract

In 2017, the European Commission published Regulation 2017/2158 establishing mitigation measures and benchmark levels to reduce acrylamide in foods. Acrylamide was determined in seventy potato crisp samples commercialized in Spain. The aim was to update knowledge about the global situation in the snack sector and evaluate the effectiveness of mitigation strategies applied, especially since the publication of the Regulation. Results were compared with data previously published in 2004, 2008, and 2014, assessing the evolution over recent years. Average acrylamide content in 2019 (664 µg/kg, range 89–1930 μg/kg) was 55.3% lower compared to 2004, 10.3% lower compared to 2008 and practically similar to results from 2014. Results support the effectiveness of mitigation measures implemented by Spanish potato crisp manufacturers. However, 27% of samples exhibited concentrations above the benchmark level established in the Regulation (750 μg/kg), which suggests that efforts to reduce acrylamide formation in this sector must be continued. Besides the variability seen between samples, acrylamide significantly correlated with the color parameter a*, which enables discrimination of whether potato crisps contain above or below benchmark content. The calculated margin of exposure for carcinogenicity was below the safety limit, which should be considered from a public health point of view.

## 1. Introduction

Acrylamide is a chemical process contaminant formed when foods containing free asparagine and reducing sugars are cooked at temperatures above 120 °C in low moisture conditions [1]. It is identified by the International Agency for Research on Cancer as being probably carcinogenic to humans (group 2A) [2]. In 2015, the Expert Panel on Contaminants in the Food Chain (CONTAM) of the European Authority on Food Safety (EFSA) concluded that the presence of acrylamide in foods potentially increases the risk of developing cancer for consumers in all age groups [1]. Among the eleven food categories evaluated, potato fried products are generally the main contributor to total dietary acrylamide exposure [1].

There are several initiatives aiming to mitigate acrylamide formation in processed foods and its consequent acrylamide exposure and health concern. In the food processing sector, FoodDrinkEurope developed the so-called “Acrylamide Toolbox”. This collects and updates the most effective technological strategies and recommendations in the food processing chain to mitigate the formation of acrylamide [3]. The Food and Drug Administration (FDA) also published guidance for acrylamide mitigation in the food industry to bring acrylamide levels down [4]. In parallel, the European Commission issued indicative values for the presence of acrylamide in foods in 2011 and 2013, based on EFSA monitoring data from 2007–2008 [5,6]. Subsequently, on 20 November 2017, the European Regulation (EU) 2017/2158 was published, establishing mitigation measures and benchmark levels for reducing the presence of acrylamide in food [7]. Although benchmark levels are not regulatory limits or safety thresholds, they serve to provide reference values for the industry. In the case that a product exceeds these values, the manufacturer should address the problem and apply relevant mitigation strategies in order to decrease acrylamide levels in the food. Focusing on potato-based products, including French fries and potato crisps, recommendations to mitigate the formation of this contaminant include selecting suitable potato varieties, controlling potato storage and transport, monitoring recipe, and process conditions, and providing information to end users about adequate cooking practices. For potato crisps, the first indicative value for acrylamide was set at 1000 µg/kg [5,6], though subsequent revisions have reduced this benchmark level to 750 µg/kg [7].

Control of any step in the manufacturing process of fried potatoes is especially important to reduce exposure to acrylamide. Variations in total dietary exposure to this contaminant of up to 80% can be expected in fried potatoes, depending on the conditions of potato frying [1]. As a result of the commitment of the potato snack sector to follow indications stipulated in the “Acrylamide Toolbox”, various studies have reported the successful application of acrylamide mitigation strategies in potato snacks over recent years. Powers et al. [8] described a statistically significant downward trend in mean acrylamide levels in potato crisps in Europe from 763 µg/kg in 2002 to 358 µg/kg in 2011 (a decrease of 53%). The same authors published an updated evaluation in 2016, describing mean concentration to be 412 µg/kg [9]. While these data represent a 46% reduction since 2002, a leveling off has been observed since 2011 [9]. In a previous study, our research group observed a decrease in acrylamide levels in potato crisps in the Spanish market from 1484 µg/kg in 2004 to 629 µg/kg in 2014, representing an overall reduction of 57.6% [10]. Despite this decline, 17.5% of the samples in 2014 registered values higher than the indicative value recommended by the European Commission for potato crisps, at the time of publication. Parallel to this, our research team, together with some others, has established the existence of a direct correlation between the CIELab color parameter a* and acrylamide content in this foodstuff, proposing a* as a useful predictor of the acrylamide formation in fried potatoes [11,12,13,14,15].

In the present investigation, the acrylamide content of potato crisps marketed in Spain was re-evaluated one year after introduction of the Regulation with a view to investigate the current state of the snack food sector and degree of compliance with the Regulation. Results were considered alongside results produced during the period 2004 to 2014. Additionally, suitability of the CIElab parameter a* for predicting acrylamide levels in potato crisps was explored.

## 2. Material and Methods

### 2.1. Chemicals and Materials

Acrylamide standard (99%), potassium hexacyanoferrate (II) trihydrate (98%, Carrez-I), and zinc acetate dehydrate (>99%, Carrez-II) were obtained from Sigma (St. Louis, MO, USA). ^13^C_3_-labelled acrylamide (99% isotopic purity) was obtained from Cambridge Isotope Laboratories (Andover, MA, USA). Formic acid (98%), methanol (99.5%) and hexane were from Panreac (Barcelona, Spain). Deionized water was obtained from a Milli-Q Integral 5 water purification system (Millipore, Billerica, MA, USA). All other chemicals, solvents and reagents were of analytical grade. Reversed-phase Oasis-HLB cartridges (30 mg, 1 mL) were from Waters (Mildford, MA, USA). Syringe filter units (0.45 µm and 0.20 µm, cellulose) were purchased from Análisis Vínicos (Tomelloso, Ciudad Real, Spain).

### 2.2. Samples

Seventy commercial potato crisps from 33 different producers were purchased in several Spanish supermarkets in February 2019. Of the producers, 11 supplied the present study with more than one brand, whilst the remaining 22 supplied a single brand only. Samples containing different flavorings and/or added spices were excluded from sampling in order to avoid bias during data interpretation. Potato crisps were classified according to producer, type of frying oil (sunflower oil, olive oil, and other/undefined oil), type of cut (smooth, wavy), oily appearance (oily, semi-oily, non-oily), and type of container (light protected, partly-light protected, non-light protected). Potato crisps (100–280 g, according to the size of the bag) were mixed and ground to ensure homogeneous distribution of potential hotspots. A portion (ca. 80 g) was distributed into two containers and stored under vacuum and light protected conditions at 4 °C, until analysis.

### 2.3. Determination of CIELAB Color

Color measurements were made at room temperature using a HunterLab Spectrophotometer 150 CM-3500D colorimeter (Hunter Associates laboratory, Stamford, CT, USA). Three independent measurements of a* (redness), b* (yellowness), and L* (lightness) parameters were carried out on different areas of the ground potato crisps in order to consider the non-homogeneous distribution of color within the same batch of the product. E index was calculated according to the following equation: E = (*L*^2^ + *a*^2^ + *b*^2^)^1/2^. Equipment was calibrated with a standard calibration white plate CR-A43 (L*/93.80, a*/0.3156, b*/0.3319).

### 2.4. LC-ESI-MS-MS Determination of Acrylamide

Acrylamide was determined in potato crisp samples as described by Mesias and Morales [10] and based on the ISO:EN:16618:2015 method. The recovery rate of acrylamide spiked to the samples was between 90 and 106%. Relative standard deviations (RSD) for precision, repeatability, and reproducibility of analyses were calculated as 2.8%, 1.2% and 2.5%, respectively. The limit of the quantitation was set at 20 µg/kg, complying with performance criteria set by EU Regulation 2017/2158 [7]. Accuracy of the results was demonstrated for potato crisps and pre-cooked French fries in four proficiency tests launched by the Food Analysis Performance Assessment Scheme (FAPAS) program, yielding a *z*-score of –0.2 (Test 3071, Feb–March 2017), −0.3 (Test 3080, Feb–March 2018), 0.0 (Test 3085, Sep–Oct 2018), and 0.3 (Test 3089, Feb-2019). Results of acrylamide were expressed as µg/kg of sample. Analysis was done in duplicate.

### 2.5. Dietary Exposure Assessment

Categories of dietary exposure to acrylamide from potato crisps were estimated by combining data for total per capita consumption of potato crisps (1.34 kg/person/year) established by the Spanish Ministry of Agriculture, Food and Environment [16], and acrylamide content of the samples. An average body weight (bw) of 70 kg was used to estimate total daily intake of acrylamide from potato crisps for the total population and expressed as µg/kg bw/day.

The margin of exposure (MOE) approach was applied to carry out risk assessment for acrylamide provided by potato crisps. MOE was calculated as the BMDL value divided by the respective total acrylamide intake, considering 430 μg/kg bw/day as the BMDL_10_ value for neurotoxicity (peripheral nerve axonal degeneration in male rats) and 170 μg/kg bw/day for carcinogenicity (harderian gland adenocarcinomas in mice). This is dictated in the EFSA opinion report on acrylamide [1].

### 2.6. Statistical Analysis

Statistical analyses were performed using SPSS version 23.0 (SPSS Inc., Chicago, IL, USA). Data were expressed as mean ± standard deviation (SD). Student *t*-test and analysis of variance (ANOVA one-way) followed by the Fisher’s test were used to identify the overall significance of differences between variables. Homogeneity of variances was determined using the Levene test. Relationships between the different variables were evaluated by computing Spearman’s linear correlation coefficients. The significance of all statistical parameters was evaluated at the level of *p* < 0.05.

## 3. Results and Discussion

### 3.1. Nutritional Composition of Potato Crisps

Table S1 depicts the average nutritional composition of potato crisps as declared by the manufacturer. Energy values (439–589 kcal/100 g) were close for all samples, with higher differences being observed in relation to other parameters. Total fat content ranged between 13.2 and 40.7 g/100 g, with saturated fat being between 1.4 and 6.1 g/100 g. Carbohydrates exhibited a mean value of 50.1 g/100 g (38.0–72.1 g/100 g) and sugar content was 0.6 g/100 g (0.1–4.7 g/100 g). Fiber showed values from 0.5 to 7.7 g/100 g, and protein values ranged from 1.0 to 7.8 (maximum) g/100 g. The maximum salt level was 1.7 g/100 g, although 3.0% of samples claimed to be ‘no added-salt’.

### 3.2. Acrylamide Levels in Potato Crisps

Seventy (non-flavored) classical potato crisps commercialized in Spain coming from 33 different snack producers were collected during February 2019 and analyzed for acrylamide. All samples showed an acrylamide content greater than the LOQ, ranging from 89 to 1930 µg/kg (Table 1). Mean, median, and 95th percentile were 664 µg/kg, 569 µg/kg, and 1576 µg/kg, respectively. These values were higher than those reported by the EFSA for the category of potato crisps made from fresh potatoes (mean value: 392 µg/kg, 95th percentile: 949 µg/kg, *n* = 31467) in European countries [1]. However, results were in line with information provided by other authors in recent years. Hai et al. [17] found levels ranging from 25 to 1620 µg/kg in potato chips from Hanoi (Vietnam), whilst Kafouris et al. [18] described an average value of 642 µg/kg in potato crisps from Cyprus (10–2193 µg/kg). Higher concentrations have been reported in potato crisps from Italy (173–3444 µg/kg, average value: 1162 µg/kg) [19] and lower value (mean value 475 µg/kg) have been described for potato crisps collected from several Portuguese local markets [20].

**Figure 1:**
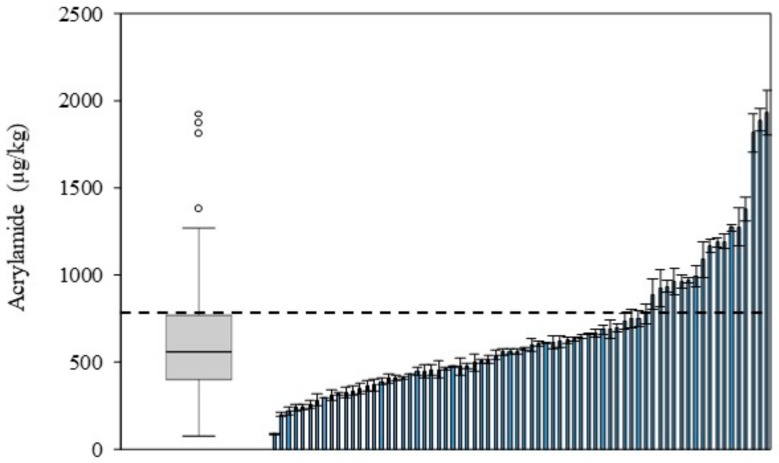
Box and whisker plot and distribution graph for the acrylamide content in Spanish potato crisps. Symbols: ◦ outliers. The dotted line indicates benchmark level (750 µg/kg) established by the European Commission [7].

Figure 1 shows acrylamide distribution in the seventy measured samples of potato crisps. Data exhibited high variability in acrylamide content between samples, in accordance with previously published results [10]. Four outliers were identified in the global distribution. As explained by Mesias and Morales [10], the 90th percentile is identified as the signal value of the dataset. Samples found with values higher than this should be especially evaluated in order to mitigate acrylamide formation. Nevertheless, nineteen of the potato crisps analyzed exceeded benchmark level for acrylamide as established by the regulation for this foodstuff (750 µg/kg) [7].

Seasonality of the potato tuber is a critical factor in strategies for acrylamide reduction since it has a great influence on levels of acrylamide precursors. Powers et al. [8] reported levels ranging from 528 µg/kg in potato crisps collected in the first six months of the year to 372 µg/kg in those collected during the second six months. Additionally, acrylamide precursor levels will depend on post-harvest storage conditions due to inadequate temperature control—for instance enabling temperatures to fall below 10 °C will increase the degradation of starch, whilst increasing the content of reducing sugars [21,22], a part of the potato variety and tuber storage time [23]. In the present study, potato crisp samples were collected in February. This is the most unfavorable period for potato producers in Spain as it is very likely that the raw material comes from stored potato. This contains higher levels of reducing sugars, thus leading to higher levels of acrylamide during frying.

Besides the nutritional composition, additional information was considered in order to evaluate the possible relationship between different processing conditions for potatoes and their acrylamide content. Thus, samples were grouped according to the type of frying oil (sunflower oil, olive oil, and other or undefined oil), type of cut (smooth and wavy), oily appearance (oily, semi-oily, and non-oily), and type of container (light- protected, partly-light protected, and non-light protected) (Table 1). Acrylamide levels were not directly affected by the type of frying oil or with the extent to which the container was light protected. Regarding the type of cut, wavy samples displayed a higher mean acrylamide concentration (786 µg/kg, *n* = 11) than smooth potato crisps (642 µg/kg, *n* = 59). These findings were not significant due to sample variability and potentially due to the unequal number of samples found for each category. Although the type of cut involves different dimensions and a potentially different acrylamide content in potato crisps, Powers et al. [9] did not observe significant differences in the acrylamide content of thick and ridge/wave potato crisps in a European sample. In our previous study, the accumulation of visually detected oil drops on the crisp’s surface was related to higher acrylamide levels [10]. In the present study, although significant differences (*p* < 0.05) were again observed with respect to the presence of oil drops on the container (Table 1), it was not possible to establish any relationship with this data.

**Figure 2:**
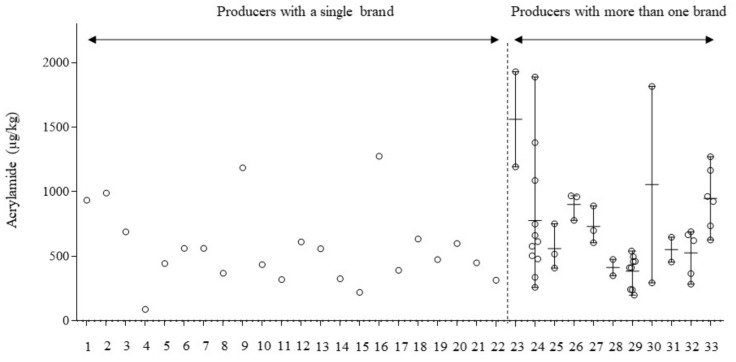
Acrylamide content in potato crisp samples from the Spanish market, grouped according to their producers (*n* = 33). Among them: producers with a single brand (*n*=22) and producers with more than one brand (*n*=11). Circles represent individual values; horizontal lines represent mean values and vertical lines represent the range for each producer.

Samples were also grouped according to the producer (Figure 2). Acrylamide content varied greatly between the 33 producers. Mean acrylamide content ranged from 89 µg/kg to 1561 µg/kg between different producers. Eight of the producers had mean values exceeding the acrylamide benchmark value set by the European regulation, especially producer 23 (1561 µg/kg) and 30 (1055 µg/kg). Nearly one-third of the producers provided more than one brand for this study. Large acrylamide variations were also observed within the same producer; for instance, samples from producer 30 ranged from 295 µg/kg to 1815 µg/kg. High variability in acrylamide content of potato crisps suggests differences in the raw material (potato variety and post-harvest storage) and processing conditions applied. Although this information is not available, it is assumed that potato crisp producers marketing a unique brand are small companies with a nearby geographical distribution of their product, in contrast to large companies marketing several brands throughout the country. From a managerial point of view, large snacking companies would have more resources to implement mitigation strategies; for instance, they are more likely to be able to select the most appropriate potato tuber. However, only four of the 22 small potato crisp producers exceeded the benchmark value for acrylamide.

### 3.3. Acrylamide–Color Relationship in Potato Crisps

**Figure 3:**
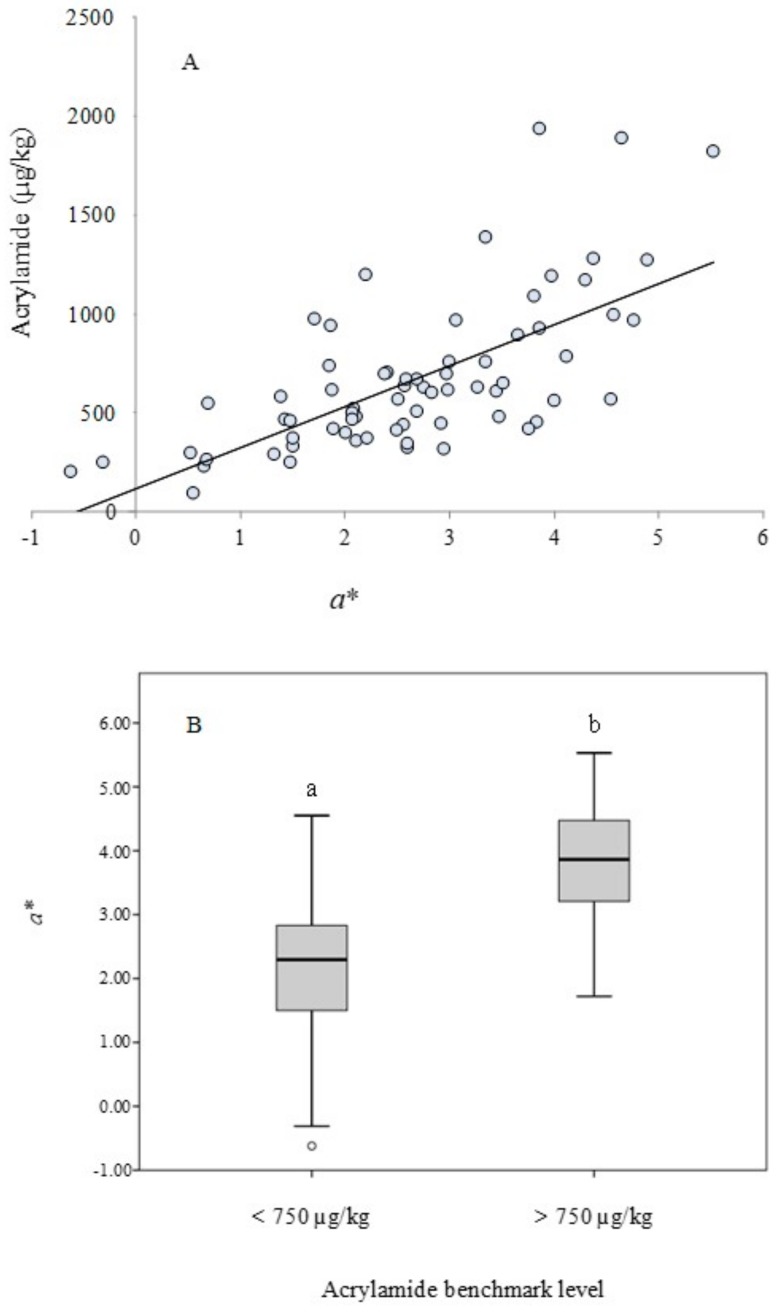
Relationship between color and acrylamide content (µg/kg) in potato crisps marketed in Spain. Linear regression between acrylamide content and the color parameter a* (r^2^ = 0.6736, *p* < 0.0001) (**A**) and box-and-whisker plot of color parameter a* according to the acrylamide levels using the benchmark level as the threshold (750 µg/kg) (**B**).

One of the critical factors to establish the end-point of frying is the color of potato chips [24]. Samples in the present study were characterized according to the CIELab color scale. Mean ± standard deviation, minimum, and maximum values for a*, b*, L*, and E are presented in Table 2. Again, a huge variability was observed between the samples, with values ranging from –0.62 to 5.53 for a*, from 16.08 to 29.22 for b*, from 50.04 to 68.01 for L*, and from 53.74 to 72.24 for E. A significant correlation was found between acrylamide and L* (r^2^ = −0.2889, *p* = 0.0153) and E (r^2^ = −0.2511, *p* = 0.0360), indicating that browning and lower luminosity in potato chips led to greater formation of this contaminant. The correlation with b* was not found to be significant; however, a strong relationship was displayed with a* (r^2^ = 0.6736, *p* < 0.0001) (Figure 3A). Two essential factors would explain that the curve does not go through zero. On the one hand, the color parameter a* in the fried potato has not been corrected by the color in the raw potato. On the other hand, the contribution to the color of the frying oil should be also considered since nearly 30% of the final product is oil that affects the color. However, even considering this limitation, the relationship is strongly significant. Previous studies have already reported a direct correlation between a* and acrylamide in this foodstuff; thus, a* has been proposed as a useful predictor of acrylamide formation in fried potatoes [11,12,13,14,15]. Besides the high variability according to sample type, producers, and acrylamide level, our results pointed out that the a^*^ parameter is capable of discriminating (*p* < 0.0001) between samples that contain above and below benchmark level of acrylamide (750 µg/kg) (Figure 3B).

### 3.4. Evaluation of Acrylamide Exposure from Spanish Commercialized Potato Crisps

Exposure to acrylamide from potato crisps in the Spanish population was estimated considering total data per capita consumption of this foodstuff as indicated by the Spanish Ministry of Agriculture, Food and Environment (1.34 kg/person/year) [16], and acrylamide content in present samples. Considering the minimum and maximum acrylamide content in the overall sample, exposure to this contaminant was seen to range between 0.33 and 7.09 µg/person/day, with a mean value of 2.44 µg/person/day. Assuming an average body weight (bw) of 70 kg, mean daily intake of acrylamide from potato crisps for the total population was estimated to be 0.035 µg/kg bw/day, ranging from 0.005 to 0.101 µg/kg bw/day. Median values matched with previous estimations made in 2015, in which average contribution of potato crisps to dietary acrylamide exposure in Spain was also calculated to be 0.035 µg/kg (range: 0.006–0.12 µg/kg bw/day) [10].

Acrylamide exposure estimated in the present assay was similar to that observed for other countries in studies with different population sectors (Table 3). The average value was similar to the median reported in adults from Italy [19], higher than mean exposure in adults from Belgium [25] and much lower than in the general population from Denmark [26]. Adolescents generally present greater exposure [18,19,25], except those from Poland [27], with median values being close to the mean described in the present study. In contrast, elderly and very elderly populations have shown lower acrylamide exposure due to lower consumption of potato crisps [19].

Risk characterization for acrylamide in potato crisps was conducted taking MOE values of 125 and 10,000 as values indicating no concern for neurotoxicity and carcinogenicity in people, respectively [1]. As mentioned above, MOE was calculated as the BMDL value divided by respective total acrylamide intake; 430 μg/kg bw/day was considered as the BMDL_10_ value for neurotoxicity, and 170 μg/kg bw/day as the value for carcinogenicity, as dictated in EFSA opinion on acrylamide [1]. In this respect, mean MOE value of 12,348 was obtained for neurotoxicity (range: 92,122–4,248 for minimum and maximum exposure). Even the maximum value was above the safety limit of 125, which indicates no health concern. In contrast, when comparing with the BMDL_10_ for carcinogenicity, a mean value of 4882 was calculated (range: 36,420–1,679 for minimum and maximum exposure). In this case, MOE was below the safety limit of 10,000, suggesting that it should be considered from a public health point of view. This remarkable outcome is in line with data reported in several studies [1,19,27].

### 3.5. Evolution of Acrylamide Levels in Spanish Commercialized Potato Crisps in Recent Years

**Figure 4:**
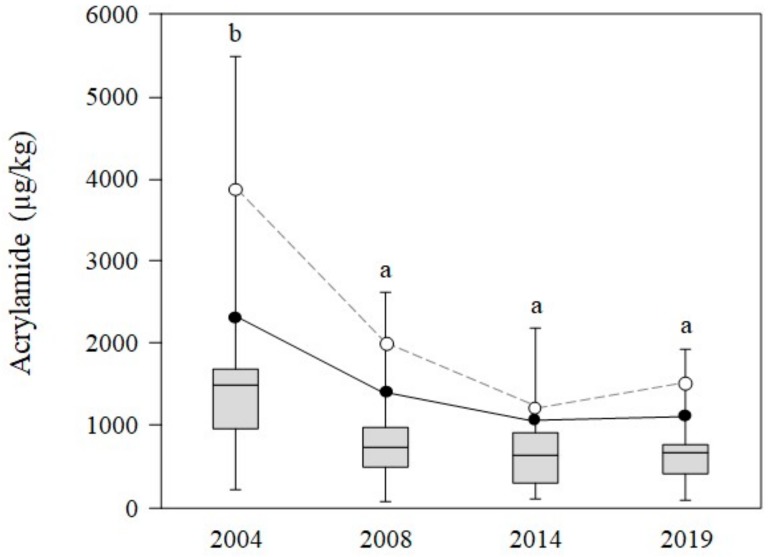
Temporal patterns for acrylamide content (µg/kg) in Spanish potato crisps sampled from 2004 to 2019. 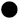 represents 90th percentile, 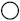 represents 95th percentile.

Acrylamide content in commercialized potato chips analyzed in this study was compared with results previously obtained by the same research group in 2014 [10], 2008 [28], and 2004 [29]. Mesias and Morales [10] showed a clear downward trend in acrylamide content of potato crisps marketed in Spain over the last 10 years. Mean values decreased from 1484 µg/kg in 2004 to 629 µg/kg in 2014, representing an overall reduction of 57.6%. Analyzing the whole period monitored between 2004 and 2019, the overall reduction was 55.2%. Statistical analysis revealed a significant reduction in the period 2004–2008 (Figure 4); however, this has since been followed by a period of leveling off, with no significant differences between mean acrylamide levels for 2008 (740 µg/kg), 2014 (629 µg/kg), and 2019 (664 µg/kg) being seen. Similar observations were displayed in the 90th and 95th percentiles. Downward trends were also seen in these values from 2004 to 2019 (Figure 4). 90th percentile values, considered as signal values, decreased from 2270 µg/kg in 2004 to 1377 µg/kg in 2008, and 1136 µg/kg in 2014, slightly increasing back up to 1187 µg/kg in 2019. Similarly, 95th percentile values decreased from 3805 µg/kg in 2004 to 1981 µg/kg in 2008, and 1233 µg/kg in 2014, increasing again to 1576 µg/kg in 2019. These findings are in agreement with results reported by Powers et al. [9]. These authors evaluated the trend in acrylamide concentration in potato crisps in Europe from 2002 to 2016, describing a 46% reduction overall throughout the period. Mean values decreased from 763 µg/kg in 2002 to 412 µg/kg in 2016. However, as in the present study, there were significant reductions between 2002 and 2011, but, since then, a leveling off has been observed, with means being even slightly greater in recent years. Thus, the year when the lowest content of acrylamide was achieved was 2011. In our case, lowest concentrations were observed in 2014.

Similarly, Claeys et al. [25] also reported a significant decrease in mean acrylamide levels in potato crisps from the Belgian market, when comparing the periods 2002–2007 and 2008–2013 (average reduction 38%). In this case, concentrations, 95th percentile values, and maximum levels decreased from 609 µg/kg (mean), 1500 µg/kg (95th), and 3200 µg/kg (maximum) in the period 2002–2007 to 375 µg/kg (mean), 725 µg/kg (95th) and 1300 µg/kg (maximum), respectively, in the period 2008–2013.

As mentioned before, 17.5% of samples taken in 2015 registered higher values than those recommended by the European Commission that year (1000 µg/kg), whilst, in the present study, 27% of analyzed potato crisps exceeded the updated benchmark value (750 µg/kg). Results illustrate the need to reduce levels of acrylamide in potato crisps since concentrations have not yet been adapted to the new recommendations. In recent years, acrylamide reduction measures in potato-based products in the acrylamide toolbox have been proposed and updated [3], as has industry guidance regarding acrylamide in foods reported by the Food and Drug Administration [4]. In addition, risk management measures for acrylamide were included and revised in the European Regulation [7].

## 4. Conclusions

Although it has been demonstrated that mitigation strategies have been successfully applied to Spanish industrial potato crisps in recent years, the greatest reduction in acrylamide levels was achieved during years close to the identification of acrylamide in foods as a health concern. Following this, declining trends have ceased and nearly one-third of samples fail to meet benchmark value established by the current European Regulation. Thus, potato crisp manufacturers must continue efforts to reduce acrylamide formation in this foodstuff in order to reach levels as low as reasonably achievable, whilst also considering the potentially pending downward revision of the benchmark level. This new study corroborates the suitability of the color parameter a* for helping producers to control the quality of potato crisps introduced into the market, so that they meet quality standards indicated by the European Regulation concerning acrylamide levels.

## Figures and Tables

**Table 1:** Statistical analysis of the acrylamide levels (µg/kg) of different potato crisp categories.

Category	Mean ± SD	Median	Minimum	Maximum	95th	*n*
Total	664 ± 387	569	89	1930	1576	70
*Type of oil*						
Sunflower	689 ± 387a	627	199	1930	1271	41
Olive oil	624 ± 333a	560	89	1381	1280	20
Other or undefined oil	642 ± 524a	459	243	1815	1563	9
*Type of cut*						
Smooth	642 ± 369a	561	89	1889	1286	59
Wavy	786 ± 473a	577	315	1930	1548	11
*Appearance*						
Oily	614 ± 287a	541	241	1275	1212	35
Semi-oily	886 ± 592b	656	221	1930	1102	12
Non-oily	626 ± 368ab	561	89	1815	1838	23
*Light protection*						
Light protected	695 ± 410a	599	199	1930	1650	47
Partly-light protected	587 ± 332a	539	89	1381	750	18
Non-light protected	653 ± 385a	541	259	1271	1349	5

Different letters in the same column mean significant differences within the same category (*p* < 0.05). SD: standard deviation. 95th: 95 percentile.

**Table 2:** CIELab parameters for potato crisps.

CIELab Parameter	Mean ± SD	Minimum	Maximum
a*	2.64 ± 0.13	-0.62	5.53
b*	24.75 ± 0.24	16.08	29.22
L*	56.97 ± 0.27	50.04	68.01
E	62.21 ± 0.38	53.74	72.24

SD: standard deviation

**Table 3:** Dietary acrylamide exposure and contribution of fried potatoes in different studies, countries, and population groups.

Country	Total Intake (µg/kg bw/day)	Potato Chips/Crisps (%)	Partial Contribution (µg/kg bw/day)		Age (years)	Reference
			Median^2^ or Mean^3^	95^th^		
Italy	n.a.^1^	n.a.^1^	0.070^2^	0.387	Toddlers	[19]
			0.112^2^	0.238	Other children	
			0.075^2^	0.201	Adolescents	
			0.030^2^	0.097	Adults	
			0.004^2^	0.029	Elderly	
			0.001^2^	0.019	Very elderly	
Poland	0.09	50	0.045^2^	0.500	Teenager girls	[27]
	0.13	34	0.044^2^	0.570	Teenager boys	
Denmark	0.31	46^4^	0.143^3^	0.382	General population	[26]
Cyprus	0.8	14	0.112^3^	0.252	Adolescents	[18]
Belgium	0.72	5.9	0.042^3^	0.143	Children	[15]
	0.48	18.8	0.090^3^	0.297	Adolescents	
	0.33	5.8	0.019^3^	0.063	Adults	

^1^ n.a.: not available. ^2^ Median value. ^3^ Mean value. ^4^ Fried potatoes including French fries

## Supplementary Materials

The following are available online at https://www.mdpi.com/2304-8158/9/2/247/s1, Table S1. Average nutritional composition of the whole dataset of potato crisps as provided by the manufacturer. Data are expressed as mean ± standard deviation (SD) per 100 g of sample
