# Supplementary material for: How Far is the Spanish Snack Sector from Meeting the Acrylamide Regulation 2017/2158?
Source: Foods. 2020 Feb 24;9(2):247. doi: 10.3390/foods9020247 (PMC7073576; doi:10.3390/foods9020247)
Supplement: Supplementary file 1 [file foods-09-00247-s001.pdf]

How far is the Spanish snack sector to meet the acrylamide regulation 2017/2158?

Marta Mesias*, Aouatif Nouali, Cristina Delgado-Andrade, Francisco J Morales

SUPLEMMENTARY

Table S1. Average nutritional composition of the whole dataset of potato crisps as provided by the manufacturer. Data are expressed as mean ± standard deviation (SD) per 100 g of sample

	**Mean ± SD**	**Minimum**	**Maximum**
Energy	536 ± 25	439	589
Total fat	33.8 ± 4.5	13.2	40.7
Saturated fat	4.1 ± 1.0	1.4	6.1
Carbohydrates	50.1 ± 5.0	38.0	72.1
Sugars	0.6 ± 0.6	0.0	4.7
Fibre	4.3 ± 1.9	0.5	7.7
Proteins	6.2 ± 1.1	1.0	7.8
Salt	0.9 ± 0.5	0.0	1.7
